# Anterior interosseous to extensor carpi radialis brevis nerve transfer for wrist extension in brachial plexus birth injury

**DOI:** 10.1007/s00381-026-07460-0

**Published:** 2026-09-14

**Authors:** Alejandro J. Friedman, Brian Pettitt, Megan Gotlieb-Horowitz, Lynn Ann Forrester, Steven M. Koehler

**Affiliations:** 1 Division of Hand Surgery, Department of Orthopaedic Surgery, Montefiore Medical Center/Albert Einstein College of Medicine, 1250 Waters Place, Tower One, 11th Floor, Bronx, NY 10461 USA; 2 Division of Plastic and Reconstructive Surgery, Department of Surgery, Montefiore Medical Center/Albert Einstein College of Medicine, Bronx, NY USA

**Keywords:** Brachial plexus birth injury, Nerve transfer, Wrist drop, Wrist extension, Anterior interosseous

## Abstract

Brachial plexus birth injuries (BPBI) affecting the C5 to C8 nerve roots commonly impair wrist extension, making its restoration a key goal of reconstruction. However, children with persistent wrist extension paralysis have limited surgical options, particularly in the setting of extensive plexus injury. Notably, infants with C5-C8 injuries often lose wrist extension while preserving median nerve function due to intact T1 contributions. We report two infants in whom wrist extension was successfully restored using an anterior interosseous nerve-to-extensor carpi radialis brevis (AIN-ECRB) nerve transfer.

## Introduction

Brachial plexus birth injuries (BPBI) affecting the inferior cervical nerve roots and lower trunk often compromise distal upper limb function. Wrist extension requires intact radial nerve contributions from C8; therefore, children with C5-C7 injury often demonstrate preserved wrist extension [1–4]. Infants with C5-C8 injuries typically lose wrist extension but retain median nerve function because of preserved T1 contributions. Median nerve-innervated muscles remain functional in many C5-C8 injuries, including the flexor carpi radialis (FCR), pronator teres (PT), anterior interosseous nerve (AIN) innervated muscles, flexor pollicis longus (FPL), flexor digitorum profundus (FDP) to the index and middle fingers, and the pronator quadratus (PQ) [4–7]. Consequently, branches of the median nerve represent reliable donor options for patients with four-root injuries [7].

Restoration of wrist extension is a critical objective in BPBI reconstruction as it facilitates effective finger flexion through the tenodesis effect and is essential for optimal grip strength. However, children with persistent paralysis of wrist extension face limited reconstructive options in the setting of extensive brachial plexus injury. When T1 powers finger extensors without functioning wrist extensors, patients develop wrist drop with associated loss of grasp strength [8]. Additionally, the ulnar nerve is frequently weak in these cases and unreliable as a donor [1]. Thus, surgeons must weigh distal nerve transfers against delayed tendon transfers to restore hand function.

Several authors have reported successful reconstruction of wrist extension in adult brachial plexus patients via transfer of the PQ branch of the AIN to the extensor carpi radialis brevis (ECRB) branch of the radial nerve [9–12]. Despite this transfer’s success in adults and the proven efficacy of nerve transfers in BPBI, this technique has not been previously reported in pediatric patients. We present two infants in whom we successfully reconstructed wrist extension using AIN-ECRB nerve transfer.

## Case report

### Patient one

A G5P2032 mother delivered a full-term female infant via spontaneous vaginal delivery complicated by right shoulder dystocia requiring multiple maneuvers. Immediately after birth, the infant demonstrated decreased right upper limb movement and asymmetric Moro and grasp reflexes. Magnetic resonance imaging obtained on day 5 of life revealed edema and hemorrhage surrounding the brachial plexus without nerve root avulsion.

At 2 weeks, evaluation in our multidisciplinary brachial plexus clinic demonstrated a four-root palsy without Horner syndrome. No clinical improvement was observed at 2 months, and ultrasonography revealed glenohumeral dysplasia (GHD). A SupER (supination-external rotation) orthosis protocol was initiated. Although bracing prevented progression of GHD, at 3 months, Active Movement Scale (AMS) scores remained poor, with scores of 0 for shoulder abduction, flexion, external rotation, elbow flexion, and forearm supination, along with a score of 4 for wrist extension and a score of 3 for thumb and finger extension.

At 15 weeks of life, surgical exploration revealed extensive cicatrix over the scalene muscles and a large neuroma at the trunk and division level affecting four roots. Post-neurolysis testing demonstrated severely diminished intraoperative conduction across the neuroma. Given these findings, we performed nerve transfers, including spinal accessory to suprascapular nerve transfer and medial pectoral to axillary nerve transfer to restore shoulder function, and double fascicular nerve transfer in the medial arm to restore elbow flexion, as well as reduction of the shoulder. In the forearm, the AIN branch to PQ was transferred end-to-end to the radial nerve branch to the ECRB, using the technique previously described by Bertelli [10].

Meaningful clinical activation of the AIN-ECRB transfer was observed at 6 months postoperatively with an active movement scale (AMS) score of 4 for wrist extension. With continued donor-activation-focused rehabilitation, the patient demonstrated full antigravity wrist extension (AMS 7) by 7 months postoperatively (Table 1) [13].

### Patient two

This infant was born at 40 weeks to a 23-year-old G1P0 mother and weighed over 4500 g. Vaginal delivery was complicated by a prolonged shoulder dystocia (> 3 min). The patient was hypotonic, apneic, and required intubation and neonatal intensive care unit admission. The patient was diagnosed with hypoxic-ischemic encephalopathy, subgaleal hemorrhage, and seizures.

At 4 weeks of age, the patient presented to BPBI clinic. The left upper limb was normal. The upper limbs demonstrated asymmetric Moro and biceps reflexes and AMS scores of 0 for all right shoulder and elbow motions as well as wrist extension. Wrist and finger flexion were scored 7 out of 7; thumb extension reached an AMS score of 6. Shoulder ultrasound demonstrated early dysplasia. SupER bracing and therapy were initiated.

At 5 months, given persistent deficits, surgical intervention was recommended. Surgery was delayed until 8 months at the family’s request. At 8 months, the child showed no significant recovery of upper limb function and ultrasound revealed worsening GHD. Intraoperatively, a massive, non-conducting neuroma involving the roots and trunks was identified. In addition, we identified avulsions of C6 and C7. The neuroma was excised, and a branch of the medial antebrachial cutaneous nerve was harvested and used as spanning autograft to connect C5 to the anterior and posterior divisions of the upper trunk. Reconstruction of shoulder and elbow movement was performed in the same manner as patient one. Due to failure of antigravity recovery of wrist extension, we transferred the AIN branch to PQ end-to-end to the radial nerve branch to the ECRB. The patient quickly activated their nerve transfer and attained a wrist extension AMS score of 5 at 2 months postoperatively; they attained AMS 6 by 3 months. At just over 12 months of age and 4 months postoperatively, the patient achieved full range antigravity wrist extension, corresponding to an AMS score of 7  (Table 1).

**Table 1:** Clinical characteristics of patients one and two. Age and follow-up time are presented in months. Active movement scale (AMS) scores are between 0 and 7, with 4 representing active antigravity movement and 6 representing functional recovery of active movement. WE wrist extension, FF finger flexion, TF thumb flexion, WF wrist flexion

	Age	Follow-up	Preop WE	Postop WE	Preop FF/TF	Postop FF/TF	Preop WF	Postop WF
Patient One	3.42	10.26	4	7	7/7	7/7	7	7
Patient Two	8.57	2.86	3	7	7/6	7/6	7	7

## Discussion

We report the first pediatric cases of wrist extension reconstruction using AIN-ECRB nerve transfer for BPBI. C5-C8 injuries produce severe functional deficits while limiting available donor options. Although the AIN has been established as a donor for ulnar and radial nerve innervated muscles in adults, its application in infants has not previously been described [8–11].

Nerve reconstruction within the first year of life yields superior outcomes compared with tendon transfers and preserves musculature for future procedures [14]. Infants also possess marked cortical plasticity, allowing rapid motor repatterning after nerve transfer [15–17]. The AIN-ECRB transfer is considered “in-phase,” pairing forearm pronation with wrist extension, which may further facilitate neuromuscular reeducation. Given the insertion of the ECRB on the 3rd metacarpal, the presence of centralized wrist extension (as opposed to radially deviated wrist extension that may result from firing of the extensor carpi radialis longus) provided evidence of reinnervation of ECRB rather than proximal reinnervation of ECRL from neurolysis or grafting. In addition, the timeline of early recovery supports recovery from nerve transfer rather than from grafting.

Donor site morbidity is an important consideration in nerve transfers, and the combination of PQ denervation and reinnervation of the biceps could, in theory, result in a neuromuscular imbalance. We did not appreciate over-supination in our patients over the follow-up period, and the cohort reported by Bertelli et al. demonstrated full retention of pronation range of motion in roughly two-thirds of patients and zero reported instances of postoperative supination contracture after AIN to ECRB transfer [1].

Both patients demonstrated early and meaningful recovery of wrist extension. These results suggest that AIN-ECRB nerve transfer is a viable option for selected infants with BPBI. This report is limited by its small sample size and short follow-up period, as well as the lack of goniometric or functional strength data, in part due to the age of our patients. Larger studies are needed to define indications, durability, and long-term functional outcomes for this patient population.

## Data Availability

All available and pertinent deidentified patient data has been presented in this case report. To protect patient privacy, other patient data is restricted, though the authors will review reasonable requests for further data.
